# Concise Review: Mesenchymal Stem Cell Therapy for Pediatric Disease: Perspectives on Success and Potential Improvements

**DOI:** 10.5966/sctm.2015-0427

**Published:** 2016-09-13

**Authors:** Christopher R. Nitkin, Tracey L. Bonfield

**Affiliations:** ^1^ Division of Neonatology, Rainbow Babies and Children's Hospital, Cleveland, Ohio, USA; ^2^ Division of Pulmonology, Rainbow Babies and Children's Hospital, Cleveland, Ohio, USA; ^3^ Department of Pediatrics, Case Western Reserve University, Cleveland, Ohio, USA

**Keywords:** Mesenchymal stem cells, Pediatric diseases, Bronchopulmonary dysplasia, Autism, Osteogenesis imperfecta, Graft versus host disease

## Abstract

Mesenchymal stem cells (MSCs) represent a potentially revolutionary therapy for a wide variety of pediatric diseases, but the optimal cell‐based therapeutics for such diversity have not yet been specified. The published clinical trials for pediatric pulmonary, cardiac, orthopedic, endocrine, neurologic, and hematologic diseases provide evidence that MSCs are indeed efficacious, but the significant heterogeneity in therapeutic approaches between studies raises new questions. The purpose of this review is to stimulate new preclinical and clinical trials to investigate these factors. First, we discuss recent clinical trials for pediatric diseases studying MSCs obtained from bone marrow, umbilical cord and umbilical cord blood, placenta, amniotic fluid, and adipose tissue. We then identify factors, some unique to pediatrics, which must be examined to optimize therapeutic efficacy, including route of administration, dose, timing of administration, the role of ex vivo differentiation, cell culture techniques, donor factors, host factors, and the immunologic implications of allogeneic therapy. Finally, we discuss some of the practicalities of bringing cell‐based therapy into the clinic, including regulatory and manufacturing considerations. The aim of this review is to inform future studies seeking to maximize therapeutic efficacy for each disease and for each patient. Stem Cells Translational Medicine *2017;6:539–565*

**Significance Statement**

Mesenchymal stem cells (MSCs) are the focus of great excitement for treating diseases associated with not just regeneration but also immunomodulation. This review focuses on the outcomes of MSC therapeutics in a variety of pediatric diseases. The discussion is based on how the trials took place and what can ultimately be learned from the outcomes of the studies. This review provides significant insight into learning the next steps toward developing better therapies for children with difficult‐to‐treat diseases.

## Introduction

First named in the 1980s by Arnold Caplan, mesenchymal stem cells (MSCs) and MSC‐based therapy have emerged as an extremely promising therapy in adult medicine, and, combined with a wealth of additional preclinical data, are expanding into the pediatric arena. Initial enthusiasm for MSC therapy stemmed from the possibility of tissue regeneration and organ engineering based on the ability of MSCs to differentiate into bone and cartilage 1. Although some osteogenic and chondrogenic disorders do appear to benefit directly from tissue regeneration, newer evidence suggests that MSCs instead represent “medicinal signaling cells” that secrete immunomodulatory, antiapoptotic, anti‐inflammatory, proangiogenic, promitogenic, and antibacterial factors 2. Indeed, preclinical data suggest that many of the benefits of cell‐based therapy may be obtained with use of cell‐free, MSC‐conditioned media. For example, data from our laboratory have demonstrated that MSCs and MSC‐conditioned media have similar benefits in models of cystic fibrosis 3 and asthma 4. Others have found the same in rodent models of bronchopulmonary dysplasia 5, 6.

The published literature includes many case reports and clinical trials for pediatric diseases as diverse as bronchopulmonary dysplasia, cardiomyopathy, hypophosphatasia and osteogenesis imperfecta, cerebral palsy and spinal muscular atrophy, autism spectrum disorders, and inborn errors of metabolism. There exist a number of excellent reviews on the use of MSC therapy in orthopedics 7, 8, 9, oral reconstructive surgery 10, graft‐versus‐host disease 11, 12, neurologic disorders 13, 14, 15, bronchopulmonary dysplasia 16, and cardiac disorders 17. A comprehensive listing of the published literature for stem cell therapy in pediatrics is beyond the scope of this concise review, but Table 1 includes some of the most recent studies, as well as first reports.

**Table 1:** Clinical trials of mesenchymal stem cells in pediatrics: Levels of evidence per the Oxford Levels of Evidence 2

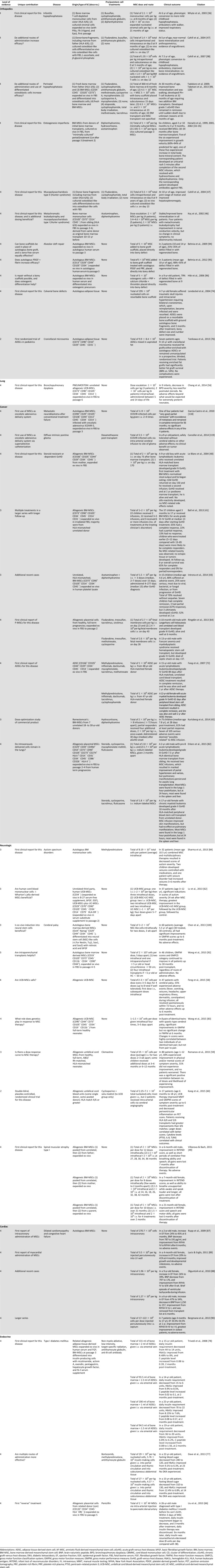

The purpose of this review is to stimulate new preclinical and clinical trials to evaluate and compare the donor, host, and cell factors contributing to MSC therapeutic efficacy. We will discuss the wide spectrum of published MSC trials for pediatric diseases, including the results from the most recent clinical studies. We highlight the marked variability in therapeutic approaches, as well as some of the unique challenges to cell‐based therapy in pediatrics. The published studies provide evidence that MSCs may successfully treat multiple pediatric diseases, but the significant heterogeneity in therapeutic approaches between studies raises new questions that must be answered with additional clinical trials. The aim of this review is to inform future studies seeking to maximize therapeutic efficacy for each disease and for each patient.

## Methods: Search Strategy

The PubMed database was searched in September 2015 by using keywords (“mesenchymal stem cell” OR “mesenchymal stromal cell”) with limits placed on human children (birth to 18 years old), including the following article types: case reports, clinical trial, controlled clinical trial, multicenter study, observational study, pragmatic clinical trial, randomized controlled trial, and twin studies. A total of 502 studies were screened for review, and preclinical studies including MSC characterization, in vitro, and nontherapeutic articles were excluded. A total of 184 articles were reviewed for inclusion.

To capture other potential articles of interest, an additional search for “stem cells” was conducted in September 2015, with limits for children (birth to 18 years of age) with date of publication in 2015, yielding 247 articles. The preclinical studies were screened out as above, as were duplicate studies, yielding 33 articles for review for inclusion. Searches for “osteogenesis imperfecta stem cell” (111 articles), “hypophosphatasia stem cell” (17 articles), “autism stem cell” (251 articles), and “cerebral palsy stem cell” (159 articles) were conducted without limits, and searches for “graft versus host disease stem cell” (2,907 articles) and “diabetes stem cell” (175 articles) were conducted with limits for children in the last 10 years. After exclusion of preclinical studies and duplicate clinical studies, 30 additional articles were reviewed for inclusion.

## Clinical Studies of MSC Therapy

We will briefly review each clinical study, placing it in the context of other studies using MSCs to treat the same disease. We will use results from each study to discuss the various types of MSCs that have been studied, delivery method and dose of MSCs, the timing of treatment, the role of ex vivo differentiation, the importance of growth conditions, and important donor and recipient factors. We will discuss each clinical study in broad terms and refer to Table 1 for specific details.

### Sources of Mesenchymal Stem Cells

The most widely accepted definition of an MSC was put forth by the International Society for Cellular Therapy in 2006 and includes three criteria: (a) MSCs must be plastic‐adherent when maintained in standard culture conditions; (b) MSCs must express CD105 (endoglin‐1), CD73 (ecto‐5‐prime‐nucleotidase), and CD90 (thymocyte antigen‐1) and lack expression of CD45 (protein tyrosine phosphatase, receptor type, C), CD34, CD14, or CD11b (integrin‐α M), CD79‐alpha or CD19 and human leukocyte antigen‐DR (HLA‐DR) surface molecules; and (c) MSCs must differentiate to osteoblasts, adipocytes, and chondroblasts in vitro 18.

More recent work suggests that additional markers may standardize definitions of MSCs as well as the multiple types of MSCs 19. Some of the sources of MSCs studied in pediatric disease include bone marrow‐derived MSCs (BM‐MSCs), Wharton's jelly or umbilical cord tissue‐derived MSCs (UC‐MSCs), umbilical cord blood‐derived MSCs (UCB‐MSCs), placenta‐derived MSCs(P‐MSCs), amniotic fluid‐derived MSCs (AF‐MSCs), and adipose tissue‐derived MSCs (ADSCs). Several reviews on the various sources of MSCs are available 20, 21, 22. Allogeneic MSCs from related or unrelated donors with various levels of HLA‐matching and autologous MSCs have been studied.

#### Bone‐Marrow Derived MSCs

BM‐MSCs are the prototypical MSC and have been the most well‐studied. Friedenstein et al. first reported the isolation and characterization of murine BM‐MSCs in the 1960s 23, 24, and Pittenger et al. reported the chondrogenic, osteogenic, and adipogenic potential of human BM‐MSCs in 1999 25. Although they are relatively easy to obtain from human iliac crest 25, 26, tibia and femur 27, and vertebrae 28, other types of MSC (e.g., umbilical cord or placenta) are more readily obtained. Additionally, BM‐MSCs are typically isolated from adults, and advanced donor age is associated with diminished proliferative capacity 29 and loss of MSC “fitness” 30. In mice, increasing donor age is associated with decreased anti‐inflammatory capacity, homing ability, and protective effect against lung injury 31. These characteristics may make the use of human adult BM‐MSCs in the pediatric population less desirable.

#### Disorders of Bone

Horwitz et al. described the first use of BM‐MSCs in pediatrics to treat patients with osteogenesis imperfecta. Also known as “brittle bone disease,” osteogenesis imperfecta is an inherited disorder of collagen characterized by skeletal deformities and propensity for fractures 32. Patients originally received allogeneic bone marrow transplants from HLA‐matched siblings, resulting in significant improvements in number of fractures and growth velocity 33, 34. However, because these benefits were not sustained, they underwent intravenous treatment with cultured BM‐MSCs 18–34 months after original bone marrow transplant 35. Bone marrow from the original transplant donors was cultured in fetal bovine serum (FBS)‐containing media, and one dose (1 × 10^6^ cells per kilogram) was administered after minimal time in culture, and a second dose (2.85–5 × 10^6^ cells per kilogram) of third‐passage MSCs was administered 8–21 days later; no immunosuppression was provided. Five of the six experienced improvement in growth velocity. Interestingly, the nonresponding patient was the only one to experience any toxicity related to MSC infusion (urticaria 5 minutes after completion of second dose, resolving after hydrocortisone and diphenhydramine treatment) and was the only patient to develop antibodies against FBS proteins. This is one of very few reported MSC‐related adverse effects. Additionally, responders demonstrated evidence of MSC engraftment (albeit low at <1%), whereas the nonresponder did not, suggesting that a systemic immune reaction may have prevented engraftment and benefit. This could imply that MSC culture with an FBS alternative would be preferable.

Whyte et al. described the use of BM‐MSCs to treat hypophosphatasia 36, a rare disorder of bone mineralization that can be lethal in the perinatal period, and for which there is no curative therapy 37. Before treatment, the 8‐month‐old patient had progressive bony undermineralization and fractures, scoliosis, and respiratory insufficiency. After receiving 2.1 × 10^8^ mononuclear cells per kilogram of fresh, T‐cell‐depleted, allogeneic BM‐MSCs from her sister (HLA 4/6 match) intravenously, she experienced generalized bone remineralization and no new fractures. However, at 21 months of age, she clinically deteriorated with multiple new fractures and was treated a second time with a “stromal cell boost.” Her sibling's BM‐MSCs were expanded ex vivo (more specific details are not provided) to 2.92 × 10^7^ per kilogram and administered intravenously, after which clinical improvement was again obtained, and by 6 years of age, she was ambulatory with a walker. Whether the success of this stromal cell boost was associated with the MSC dose, cell viability, or cell potency, or alternative patient severity has not yet been determined. No treatment‐related adverse effects were reported.

#### Graft‐Versus‐Host Disease

BM‐MSCs have been used successfully for graft‐versus‐host disease (GvHD), a disease that can complicate hematopoietic stem cell transplant, affecting the skin, gastrointestinal tract, liver, kidney, bone marrow, joint, muscle, and lungs. Conventional therapy includes immunosuppressants, such as high‐dose steroids and cyclosporine, but many patients fail to respond or become dependent on these therapies, which have numerous adverse side effects 38. A systematic review and meta‐analysis very recently published by Hashmi et al. on the use of MSC therapy in adult and pediatric steroid‐resistant acute GvHD found an overall response rate of 73% in the subgroup analysis of pediatric patients 39.

The first case study on MSC therapy for GvHD was reported for a 9‐year‐old boy by Le Blanc et al. in 2004 40. He developed treatment‐resistant grade IV GvHD after bone marrow transplant for leukemia, and his symptoms nearly resolved after initial intravenous treatment with 2 × 10^6^ cells per kilogram of ex vivo expanded BM‐MSCs from his mother. Mild GvHD recurred, and symptoms resolved after a second treatment of 1 × 10^6^ cells per kilogram, and he was alive and well 1 year after bone marrow transplant (292 days after BM‐MSC treatment). He showed no signs of *allo*‐reactivity and experienced no MSC‐related adverse events. This was the only patient in the authors’ experience who had survived such severe disease.

Ball et al. reported results of a large retrospective cohort of 37 children who received allogeneic BM‐MSCs for acute grade III–IV GvHD 41. Multiple infusions were given at the treating clinician's discretion (typically based on response and severity of disease), and, strikingly, 65% of patients had a complete response and 22% had a partial response. No obvious dose‐dependent relationship was observed, but early treatment (5–12 days after initiating steroids for GvHD) was more likely to result in complete response than late treatment (13–85 days), and complete responders had significantly better overall survival at 6 years (65% vs. 0%). No MSC‐related toxicity was observed.

In 2012, Prochymal, a commercial BM‐MSC product (remestemcel‐L), became the first stem cell product to gain approval, labeled for use in steroid‐resistant acute GvHD. A confirmatory, open‐label, prospective, multicenter trial was published in 2014, reporting that 61% of patients had at least a partial response to eight intravenous infusions of 2 × 10^6^ cells per kilogram (with four additional treatments allowed for partial responders). GvHD of the skin was the most responsive, followed by gastrointestinal tract and liver. None of the 105 serious adverse events were deemed “likely” or “definitely” related to MSC therapy, and 7 were believed “possibly related” to MSC therapy 42. These included neutropenia, tachycardia, infusion‐related reaction, respiratory distress, pulmonary hemorrhage, and hypertension. An earlier study found similar results, with 10 of 15 patients having at least a partial response after receiving three (median) infusions of 1.5 × 10^6^ HLA‐mismatched allogeneic BM‐MSCs per kilogram intravenously 43. No patients experienced treatment‐related toxicity, but three of the responders eventually developed chronic GvHD.

#### Lysosomal Storage Disease

There are preliminary data suggesting that BM‐MSCs may be helpful in treating patients with lysosomal storage disorders, such as metachromatic leukodystrophy, Hurler syndrome (mucopolysaccharidosis type I), and Hunter syndrome (mucopolysaccharidosis type II). These disorders are generally characterized by disordered growth and bony abnormalities, gradual deterioration of cognitive and motor skills, and early death from cardiorespiratory failure 44, 45. HLA‐matched BM‐MSCs expanded ex vivo in FBS administered in dose escalation of 2 to 10 × 10^6^ MSC per kilogram intravenously to children with metachromatic leukodystrophy or Hunter syndrome resulted in improvements in bone mineralization and nerve conduction velocity 46. No *allo*‐immunity was induced (as measured by ELISPOT assay), and no treatment‐related toxicities or GvHD were observed. A case of a patient with Hurler syndrome found that BM‐MSC therapy (intraperitoneal and intraosseous donor bone fragments from an HLA‐matched sister) improved joint range of motion and stabilized the patient's clinical course 47. No adverse events were reported.

#### Spinal Muscular Atrophy

Spinal muscular atrophy type I is a progressive neurodegenerative disease that manifests in the first year of life and, in its most severe forms, requires continuous ventilatory support 48. No spontaneous improvements in muscle strength have ever been reported. A recent case series reported the first use of allogeneic BM‐MSCs in three patients 49. Multiple doses (14–17) of combined intrathecal and intravenous BM‐MSCs resulted in significant improvements in muscle strength (measured by Children's Hospital of Philadelphia Infant Test of Neuromuscular Disorders score), facial expressivity, ventilator‐free breathing ability, and ability to speak. No adverse events were reported; unfortunately, improvements were lost 6–7 months after therapy was discontinued. This suggests that, although low engraftment is typically observed, the mechanism by which MSCs exert their therapeutic effect may, in certain diseases, be a maintenance effect more than a curative repair effect.

#### Umbilical Cord‐Derived and Umbilical Cord Blood‐Derived MSCs

While BM‐MSCs have been the “gold standard,” there is compelling evidence that UC/UCB‐MSCs may be a “platinum standard.” UC‐MSCs and UCB‐MSCs are both available in relatively large quantities from morally acceptable sources with collection using no painful or invasive techniques. UC and UCB may be richer sources of MSCs, based on colony forming unit‐fibroblastic efficiency, and generate MSCs with greater immunomodulatory potential than BM‐MSCs 50.

MSCs in general are immune‐evasive, lacking major histocompatibility complex (MHC) class II, but UC/UCB‐MSCs may be even more immune‐evasive than BM‐MSCs in their ability to inhibit T‐cell alloreactivity and B‐cell proliferation and to interfere with the function of antigen‐presenting cells 51. Therefore, MSCs from umbilical cord products may be usable in scenarios in which myeloablative therapy is contraindicated (e.g., in neonates), especially with the added benefit of managing GvHD 52.

#### Bronchopulmonary Dysplasia

Bronchopulmonary dysplasia (BPD) affects up to 50% of the smallest neonates (birthweight < 1,000 g) and is the most common long‐term pulmonary morbidity experienced by infants born prematurely 53, 54. Severe BPD is associated with neurodevelopmental impairment, pulmonary hypertension, cor pulmonale, and death 55.

Exciting results of the first clinical trial of MSCs in neonates for BPD were reported in 2014 56. PNEUMOSTEM, an allogeneic human UCB‐MSC product (expanded ex vivo in FBS to passage 6), was administered in a dose‐escalation study (1–2 × 10^7^ cells per kilogram) via endotracheal tube to 5‐ to 14‐day‐old extremely preterm neonates born at 27 weeks of gestation or younger. Compared with age‐matched historical controls, treated infants developed less severe BPD and exhibited reduced levels of inflammation, and no treatment‐related adverse effects were observed. An open‐label dose‐escalation trial for these fragile infants is currently enrolling in the U.S. (clinicaltrials.gov; NCT02381366). Extremely preterm neonates often require mechanical ventilation and exogenous surfactant replacement therapy, and addition of MSCs is unlikely to represent a significant burden above the current standard‐of‐care. However, the sequence of therapeutic intervention should be considered in the trial development: using an “off‐the‐shelf” allogeneic product may be more timely than using an individualized autologous product.

#### Cerebral Palsy

Cerebral palsy represents a nonprogressive spectrum of clinical features affecting motor tone and movement and is most commonly due to premature birth, affecting up to 15% of infants with birthweight less than 1,500 g 57. A retrospective case series of 47 patients found allogeneic UCB‐MSCs administered both intrathecally and intravenously to be generally safe 58. Infusion‐related adverse effects were only observed during intrathecal administration under general anesthesia; fever and vomiting were the most common, and seizures were the most serious, but all symptoms resolved spontaneously within 72 hours. Interestingly, age ≤ 10 years predicted an increased risk of experiencing an adverse event, which the authors hypothesize may be dose‐related because the same number of cells was administered regardless of patient weight or age. However, fever and vomiting could have been related to general anesthesia. No further complications arose over a 6‐month follow‐up period. An efficacy case series of 80 patients found that allogeneic, ABO/Rh‐matched, HLA‐mismatched UC‐MSCs administered intravenously resulted in 69% of patients improving muscle tone, strength, speech, memory, attention, or cognition in a dose‐dependent manner, with no worsening of symptoms 59. No treatment‐related adverse effects were reported, giving further credence to the possibility that the fever and vomiting reported earlier were indeed related to the general anesthesia and intrathecal administration. Although most studies have reported no serious adverse effects, standardized reporting should be used to facilitate comparisons between trials, particularly in pediatric patients, who may be unable to fully articulate their subjective experience.

Similar findings were reported by Kang et al. in 2015, showing functional improvement (manual motor testing, gross motor function measure, and gross motor performance measure) at 6 months for 17 patients who received allogeneic UCB‐MSCs (HLA 4/6 or greater match) intravenously for cerebral palsy 60. Interestingly, the anti‐inflammatory effect of MSCs was shown to correlate with benefit, because decreased periventricular inflammation in the ^18^F‐fluorodeoxyglucose positron emission tomography scan was observed in treated, but not control, patients, and differential effects on cytokine and Toll‐like receptor expression were seen in treated and control patients.

#### Autism‐Spectrum Disorders

The underlying pathology in autism‐spectrum disorder (ASD), such as immune system dysregulation and cerebral hypoperfusion and inflammation, are targetable with MSC therapy. Because stem cells had been used to treat other neurologic diseases, Ichim et al. proposed a trial of MSC therapy for autism in 2007 61. Lv et al. reported results of a combined UC‐ and UCB‐MSC therapy, suggesting a synergistic effect of dual therapy 62. Thirty‐seven children with ASDs received either combined allogeneic UCB‐MSCs and UC‐MSCs, UCB‐MSCs alone, or neither via intrathecal and intravenous routes. However, all groups received standard rehabilitation therapy, and group assignment was not randomized; in fact, children in the two groups who received MSCs were enrolled at a different hospital from the children who did not receive MSCs. At 24 weeks after treatment, significant reductions in symptom severity were observed (measured by the Childhood Autism Rating Scale, Clinical Global Impression scale, and Aberrant Behavior Checklist), with the greatest improvement in the combined group, followed by the UCB‐MSC group, followed by the control group. This supports a potential positive, short‐term effect of MSCs on ASD and suggests a possible dose‐dependent relationship, although center effect cannot be discounted.

#### Placenta‐Derived and Amniotic Fluid‐Derived MSCs

Placental‐ and amniotic fluid‐derived MSCs have not been extensively studied in pediatric disease, but like UC/UCB‐MSCs, they are easily obtained in relatively large quantities from ethically acceptable sources, because both placenta and amniotic fluid are typically discarded. The placenta, or fetal membrane, has historically been a “healing tissue,” having been used successfully for burn treatment and corneal surface injury 63. Collection of AF‐MSCs for culture and tissue engineering before birth may also allow for an autologous therapeutic to be administered at or shortly after birth.

#### GvHD

A report of nine patients, including two children, investigated the use of P‐MSCs from healthy, unrelated, full‐term pregnancies for acute GvHD 64. A 13‐year‐old boy underwent matched, unrelated hematopoietic stem cell transplant for Fanconi anemia and myelodysplastic syndrome, but developed grade IV GvHD. He was treated with 2.66 × 10^6^ fetal membrane cells per kilogram intravenously, which had been expanded ex vivo in FBS to passage 2. He developed seizures, but these were thought to be due to his disease and not related to MSC therapy. He experienced mild improvement, but died of septic shock 37 days after treatment.

In contrast, the other child, a 10‐month‐old male with Langerhans cell histiocytosis, developed grade III GvHD after matched, unrelated cord blood transplantation and responded well. He received P‐MSCs at a dose of 2.6 × 10^6^ per kilogram intravenously for initial GvHD and a booster dose of 2.7 × 10^6^ cells per kilogram for recurrence, and both times experienced complete remission. He was alive and well 6 months after treatment, and no adverse effects related to MSC therapy were reported.

#### Cardiac Valvular Disease

Schmidt et al. (2007) 65 carried out a fascinating proof‐of‐concept study to prenatally engineer autologous heart valves from AF‐MSCs. After obtaining AF‐MSCs from fluid obtained for amniocentesis, they induced differentiation into fibroblast‐ or endothelial cell‐like phenotypes and applied them to a biodegradable scaffold. The resultant engineered valves were not capable of withstanding normal systemic blood pressure to serve as aortic valve replacements, but were believed to be suitable as lower‐pressure pulmonary valve replacements. Of course, in vivo studies of function and long‐term durability are needed, but such results remind us not to completely discard the tissue‐engineering promise of MSCs.

#### Diabetes Mellitus

Diabetes mellitus type 1 is an autoimmune disorder characterized by insulin deficiency that can lead to diabetic ketoacidosis crises, poor healing and limb amputations, renal failure, blindness, and heart disease, with a lifelong requirement for insulin. In a novel, “reverse” treatment, a 26‐year‐old male with newly diagnosed type I diabetes mellitus was treated with P‐MSCs from his infant son's placenta 66. P‐MSCs were cultured in FBS‐containing media until passage 5, at which time 2 × 10^7^ cells were transfused into the man's pancreatic dorsal artery. Within 3 days of treatment, his basal insulin requirement began to decrease. From months 3–9, he required no insulin, but when resumed, required a much lower dose.

#### Adipose Tissue‐Derived MSCs

ADSCs are a multipotent MSC typically derived from white adipose tissue and have been reviewed recently 67, 68. Initially described by Zuk et al. in 2001 upon processing of lipoaspirate from patients undergoing liposuction, it was found that enzymatic digestion followed by cell culture resulted in a population of cells that were able to differentiate into adipogenic, chondrogenic, myogenic, and osteogenic cells 69. However, cell surface markers vary from BM‐MSCs, and the ADSC phenotype has yet to be fully defined 67, 68.

Compared with BM‐MSCs, ADSCs can be obtained in larger quantities through a less invasive harvesting procedure 70. In fact, it is estimated that the frequency of colony‐forming units of adipose tissue is 500 times higher than that of bone marrow 71. However, one concern unique to ADSCs is that adipose tissue is an endocrine organ and therefore secretes hormones such as leptin 69. Additionally, ADSCs from obese individuals may promote a proinflammatory environment, making the conventional source (i.e., liposuction aspirates) less desirable: ADSCs derived from obese individuals, but not lean individuals, induces secretion of inflammatory cytokines (e.g., interleukin‐17A [IL‐17A] or IL‐1β) from monocytes when cocultured in vitro.

#### GvHD

Fang et al. 72 reported on two children who experienced complete remission of steroid‐resistant GvHD after ADSC treatment. A 15‐year‐old boy with Philadelphia‐chromosome‐positive acute lymphoblastic leukemia developed grade IV GvHD 89 days after HLA‐matched, unrelated cord blood transplant. He was treated with 1 × 10^6^ ADSCs per kilogram from a 48‐year‐old female donor and experienced complete remission. He was discharged home 40 days after ADSC treatment, and at the time of publication, was alive and well 1 year after ADSC treatment.

The other patient, a 12‐year‐old girl with acute myeloid leukemia in first remission, developed grade IV GvHD 62 days after peripheral blood stem cell transplant from her sibling. She was treated with 1 × 10^6^ ADSCs per kilogram from a 47‐year‐old female donor, and experienced complete remission within 3 weeks. At the time of publication, she was alive and well 2 years after ADSC treatment. No adverse effects related to ADSC treatment were reported for either patient.

#### Reconstructive Surgery

Calvarial (skull) bone reconstruction is needed in several pathogenic processes, including trauma, resection of tumors or infected bone, and congenital anomalies 73. The ideal replacement material is autologous bone because of its mechanical and immunological properties, but obtaining sufficient donor bone material in children is often difficult 73, 74. Therefore, Lendeckel et al. 74 described the use of ADSCs to repair traumatic skull defects in a 7‐year‐old girl. After injury, she developed intracranial hypertension requiring bilateral craniotomies; however, the reimplanted calvarial fragments become chronically infected and were resorbed, resulting in an unstable skull. A portion of iliac crest was ground into bone fragments and placed on a resorbable scaffold, to which autologous ADSCs and fibrin glue were applied. Computed tomography scan 3 months postoperatively showed ossification and marked improvement in defect size and number. The authors speculate that growth factors produced by the bone fragments may have stimulated ADSC differentiation into osteoblasts and osteocytes.

Craniofacial microsomia is a congenital malformation of the face, second most common to cleft lip and palate, and can result in difficulties with speech, breathing, feeding, sleep, and mental health 75. Reconstruction of affected structures, ranging from the ear and orbit to the mandible and maxilla, is necessary to maintain function, and structural fat grafts are used to achieve facial symmetry and to optimize physical appearance and psychosocial well‐being. However, 30%–80% of injected fat is resorbed 75, necessitating multiple surgeries. Therefore, Tanikawa et al. 76 investigated ADSC‐enriched fat grafts in a blinded, prospective, randomized trial for facial reconstruction. Seven patients aged 9–15 years received fat grafts enriched with autologous ADSCs, compared with seven patients aged 9–27 years who received standard fat grafts. Lipoaspirate was divided, with half undergoing enzymatic digestion for isolation of ADSCs and half remaining unprocessed, to which the ADSCs were added. In the control group, the lipoaspirate alone contained 5.6 ± 10.8 × 10^5^ per ml of viable cells, and in the experimental group, the lipoaspirate alone contained 5.7 ± 5.7 × 10^5^ per ml of viable cells, which increased to 9.9 ± 8.4 × 10^5^ per ml after ADSC enrichment. On computed tomography scans 6 months postoperatively, fat graft survival by volume was 88% in the experimental group, compared with 54% in the control group, and no patient experienced any complications. Furthermore, ADSC‐enrichment of fat grafts added only 45 minutes of operative time and incurred minimal financial cost.

#### Diabetes Mellitus

Drawing on preclinical experience, the Trivedi group has perfected a technique of inducing adipose‐derived MSCs to differentiate into insulin‐producing cells 77, 78. Individuals aged 14–22 years received allogeneic ex vivo differentiated insulin‐producing ADSCs plus bone marrow; milliliters of cell suspension and not specific cell count was provided. Treated patients exhibited marked reductions in daily insulin dose (average 65% reduction) and glycosylated hemoglobin (average 1.9%), as well as increases in C‐peptide, indicative of host insulin production. Unfortunately, because less than 2% of the volume infused was insulin‐producing ADSCs (the remaining 98% was bone marrow), it is difficult to state the relative contributions of ADSCs compared with bone marrow.

### Delivery Route, Dose, and Timing of MSCs

#### Delivery Route

The optimal delivery route and dose for MSC administration has not yet been established, and will likely need to be tailored by disease. It is unknown whether systemic delivery (i.e., intravenous) or directed delivery is optimal. Placement of support structures such as bony scaffolds for orthopedic disorders, intrathecal administration for neurologic disorders, and intratracheal administration for respiratory disorders all take advantage of directed therapy. Should conditioned media or exosomal products be administered in lieu of cells, these routes may also avoid issues such as hepatic first‐pass metabolism.

#### Intravenous Delivery

Intravenous delivery (i.v.) is the most commonly used and simplest route, and allows delivery of a large number of MSCs. Except where otherwise stated, the clinical studies in this review have investigated i.v. delivery of MSCs. However, it is known that MSCs delivered i.v. can be trapped in the lungs because of cell size relative to the pulmonary vasculature, making this a potentially less preferable route for nonpulmonary diseases. A study of MSCs administered i.v. to rats found few larger MSCs (15–19 μm) passed the lungs to the systemic arterial circulation, compared with smaller, 7‐μm cells 79. In mice, viable MSCs could only be isolated from the lungs and did not home to the liver, even when liver injury was induced by ischemia‐reperfusion 80. Conversely, in two small human studies, including six pediatric patients using indium‐labeled MSCs, although most of the signal was identified in the lungs early on, greater proportions were detected in the spleen and liver after 48 hours 81, 82. Therefore, we must not rely on animal studies alone as we optimize MSC therapy for clinical use.

#### Intrathecal Delivery

Intrathecal MSC delivery has been evaluated for cerebral palsy and autism spectrum disorders and is technically feasible in most children, including premature neonates. Wang et al. reported that allogeneic UCB‐MSCs given intrathecally to eight pairs of identical twins with cerebral palsy resulted in improvements in gross motor function 83. All patients received four intrathecal infusions of 1–1.5 × 10^7^ cells 3–5 days apart. Interestingly, improvements were correlated between the two individuals of an identical twin pair, but not between pairs of twins, suggesting that response is partially related to genetic factors.

The first clinical study of MSCs for ASDs was published in 2013 84, where intrathecal administration of BM‐MSCs resulted in significant improvements in symptom severity. These children, however, also received extensive multidisciplinary therapy, making it impossible to ascertain the relative contributions of MSC therapy and behavioral therapy.

#### Intraparenchymal/Arterial

Direct therapy into the cardiac or brain parenchyma has also been described for dilated cardiomyopathy and cerebral palsy, respectively. A potential limitation to pediatric use is body weight, because some devices are simply too large or the procedure is too technically challenging. For example, cardiac catheterization is typically performed only in infants weighing at least 2.5 kg 85, limiting the patient population that meets the criteria for safe delivery. Also, the need for specially trained surgeons and anesthesiologists may limit therapy to large children's hospitals, potentially requiring families to travel to other cities or states and taking leaves of absence from work and school.

Dilated cardiomyopathy (DCM) is the most common cause for heart failure in children, and the only curative therapy is heart transplant 86. Four studies have investigated autologous BM‐MSCs for severe DCM. Rupp et al. reported the first intracoronary administration of cells in a 2‐year‐old male whose ejection fraction nearly doubled with improvement from New York Heart Association (NYHA) class IV heart failure to class I 87. A series of two similarly ill patients also reported improvement in NYHA class after intracoronary administration of MSCs, enough for one child to be removed from the transplant list 88. Additionally, BM‐MSCs administered directly into the left ventricular wall or interventricular septum were investigated in a series of eight children 89, 90, again, with a marked improvement in ejection fraction and NYHA class. No MSC‐related adverse effects were noted in any case. Spontaneous improvement of DCM is possible, but less likely with more severe disease 91, suggesting that these outcomes are truly because of MSC therapy.

The feasibility and efficacy of MSC therapy via intraparenchymal administration for cerebral palsy was investigated in an open‐label, observer‐blinded trial of 52 patients 92. Autologous BM‐MSCs were expanded ex vivo in FBS to passage 4–5, and doses of 2 × 10^7^ cells were administered. All patients received intrathecal MSCs, but older and larger patients (5 years of age or head circumference 50 cm or greater) also received an intraparenchymal treatment via stereotactic surgery. Scores of gross motor function improved in all patients, but intraparenchymal administration did not confer additional benefit. Transient hypothermia and wound pain, but no adverse events that were more serious, were observed. It would not appear that the significant risk of injury and need for pediatric neurosurgery is outweighed by any clinical benefit.

#### Intratracheal Delivery

Results of a phase I clinical trial of infants at risk of developing BPD has provided early evidence that MSC therapy may be effective 56. Infants with the greatest risk of developing BPD typically require endotracheal intubation for mechanical ventilation and surfactant replacement therapy at or shortly after birth, providing an easy route of administration for MSCs or other cell‐based therapeutics. However, current clinical practice is to remove endotracheal tubes much earlier in the infant's hospital course (often days) than has been historically practiced (weeks to months). This may limit the use of autologous MSCs because of the weeks required to generate MSCs from umbilical cord tissue or blood 93. In this case, i.v. administration of MSCs may be an acceptable alternative to intratracheal delivery because of the potential for MSCs to be “trapped” in the pulmonary vasculature, even when administered systemically (Intravenous Delivery). Therefore, questions regarding the use of MSCs as either prophylactic or therapeutic should be answered to determine the optimal route of delivery.

#### Delivery via Support Structures

Repair of cleft palates and other bony defects is conventionally performed with autologous bone or synthetic substitutes, but both have a number of disadvantages, such as the need for a second surgical site. Behnia et al. reported on the use of autologous BM‐MSCs combined with a demineralized bone matrix scaffold, with or without platelet‐rich fibrin, but they were only able to achieve approximately 50% filling of the bony defect, inadequate for universal clinical application 94, 95. In contrast, Hibi et al. achieved 79% filling without a bone scaffold, but used BM‐MSCs that were ex vivo‐differentiated into osteogenic precursors 96, suggesting that the cells are more important than their support structure.

#### Dose

The optimal dose of MSCs is unknown and is likely to vary based on the underlying disease and severity and the route of administration. The small number of subjects in trials to date makes interpretation and extrapolation difficult. Many preclinical models have demonstrated therapeutic benefit of MSCs, and animal studies can potentially guide the initial dose‐finding studies. It is tempting to associate higher doses with greater efficacy, but toxicity and a “dose ceiling” may limit very high doses. Ethically, maximizing justice in the use of this scarce, difficult‐to‐scale resource must also be considered, particularly in the adolescent or young adult patient for whom a greater number of cells may be required.

The lack of control groups or standardized doses in many reports makes dose optimization difficult, although several reports have found that “booster” doses of MSCs were needed to maintain clinical improvement. Some trials have formally evaluated dose‐response. The dose‐escalation trial of UCB‐MSCs for BPD did not find evidence of dose‐dependent toxicity, but, interestingly, a trend toward greater benefit with the lower dose was observed 56 (a similar inverse relationship was seen in the adult POSEIDON trial of BM‐MSCs for myocardial infarction 97). In contrast, administration of UC‐MSCs for cerebral palsy showed a significant positive correlation between number of doses of UC‐MSCs and likelihood of experiencing improvement 59. Finally, the meta‐analysis of MSC therapy for acute GvHD did not find response to be dose‐dependent 39.

As discussed above, combined UC‐MSC and UCB‐MSC therapy appeared to be synergistic for treatment of autism spectrum disorders 62. Interestingly, the addition of donor bone fragment implantation to provide a greater dose of cells, including ex vivo‐differentiated osteoblasts, has been investigated for hypophosphatasia 47, 98 with some promising effects, and the use of donor bone as a source of growth factors may have contributed to the benefit observed for ADSCs used for calvarial bone reconstruction 74.

#### Timing

Timing of delivery is also important: should MSCs be given prophylactically or therapeutically? In our opinion, determining the optimal source of MSCs is a key first step before optimizing timing of administration. Head‐to‐head clinical trials of multiple MSC types, including autologous and allogeneic MSCs, would begin answering this question. Choosing autologous cells could potentially limit prophylactic administration because it can take many weeks to culture MSCs from tissue sources 93. However, an “off‐the‐shelf” allogeneic product, such as PNEUMOSTEM as used in the BPD trial 56, could be administered within the first few minutes of life or within hours of diagnosis.

As diseases progress from acute to chronic, we speculate that there may be a critical “inflection point” in the clinical course when MSC therapy is most effective. For example, in a retrospective cohort of children treated with BM‐MSCs for steroid‐resistant GvHD, treatment with MSCs earlier in the disease course (5–12 days versus 13–85 days after initiating steroid therapy for GvHD) was more likely to result in a complete response (78% vs. 52%) 41.

### The Role of Ex Vivo Differentiation

Catering cell types to the specific disease suggests that some tissue engineering is likely to be required. Ex vivo expansion and differentiation appears to be important for treatment of disorders of bone, cerebral palsy, and diabetes mellitus type I. It is possible that differentiated cells are “primed” to respond to a particular organ system and generate the appropriate growth factors and cytokines necessary for repair. Alternatively, differentiated cells may more efficiently produce the factors needed for recruitment of endogenous stem cells.

#### Osteogenic Precursors

The addition of ex vivo‐differentiated osteoblastic cells appears to impact de novo bone formation. When undifferentiated MSCs were used in cleft palate repair, inadequate bone regeneration was observed, even in the presence of a bone scaffold 94, 95. However, when differentiated into osteogenic precursors, 79% bone regeneration was achieved without a bone scaffold 96. Similarly, addition of differentiated osteoblastic cells appeared to result in bone formation in hypophosphatasia 47, 98, 99.

#### Neural Precursors

The ex vivo differentiation of autologous BM‐MSCs into neural stem cell (NSC)‐like cells was found to produce a benefit in gross motor function when administered intrathecally for cerebral palsy 100. An open‐label, prospective, nonrandomized trial included 30 patients (average age 5.5 years) who underwent bone marrow aspiration from which BM‐MSCs were cultivated then induced to differentiate into NSC‐like cells with basic fibroblast growth factor and retinoic acid. A total of 1–2 × 10^7^ NSC‐like cells were infused intrathecally 3 and 6 weeks after bone marrow aspiration, and, compared with 30 matched controls, treated patients experienced significant improvement in gross motor function up to 6 months after treatment. One patient experienced increased frequency of crying that resolved spontaneously after 48 hours, but no other adverse effects were observed. The authors hypothesize that these NSC‐like cells may have differentiated into neurons or produced beneficial neurotrophic factors.

#### Insulin‐Producing Cells

As described above in Adipose Tissue‐Derived MSCs, insulin‐producing ADSCs were used to treat patients with type I diabetes mellitus with good effect 77, 78, but the relative contributions of ADSCs and bone marrow were difficult to ascertain.

### Importance of Growth Conditions

#### Cell Culture Factors

Although a mainstay of cell culture, the use of fetal bovine serum limits the scalability of stem cell therapy because of limited global supply and batch‐to‐batch variability, and it is not ideal in the development of therapeutic agents for humans, in large part because of the risk of transmitting zoonotic pathogens such as prions. Multiple xenobiotic‐free alternatives of cell culture medium supplemented with synthetic growth factors are available, but the superiority of any one product has yet to be determined, and commercial interests preclude identifying any particular combination of supplements within these proprietary formulas. Platelet concentrates are human serum‐derived products that can be used as a source of growth factors, such as vascular endothelial growth factor and platelet‐derived growth factor 101, which are released from activated platelets 102. Therefore, autologous platelet concentrates represent an attractive alternative to bovine supplements. The types of platelet concentrates can be broadly characterized as either platelet‐rich plasma (PRP) or platelet‐rich fibrin (PRF) with or without leukocytes based on by whether the preparation consists of low‐ or high‐density fibrin networks and by the presence or absence of leukocytes. Several groups have evaluated these different PRF or PRP preparations, studying growth factor concentrations 101, 102, the effect of leukocytes 103, and the variability between donors 103. Finally, recent studies comparing platelet lysate, defined xenobiotic‐free supplements, and FBS have been published 104, 105, 106, 107, 108, 109, 110, 111.

Generally, autologous platelet concentrate is preferable, but developing sufficient quantities from young patients may be limited by blood volume. The World Health Organization reviewed pediatric blood‐draw policies for research 112, finding that institutions typically limit single‐draw volume to 1%–5% of the total blood volume, equating to 5–6 ml in full‐term newborns, with volumes on the order of 30 ml, as required to generate autologous PRP or PRF, allowed only in children closer to preschool age. One option that has not been explored is the use of allogeneic PRP or PRF with autologous MSCs.

Although FBS and platelet concentrates have not been compared directly in clinical trials, a meta‐analysis of MSC therapy for adult and pediatric GvHD combined found that a greater proportion of patients responded to treatment when MSCs had been cultured in FBS, compared with human platelet lysate (76% vs. 62%) 39. For example, Introna et al. used human platelet lysate for BM‐MSCs to treat pediatric GvHD, and 67% had at least a partial response 43. Mixed results have been obtained by using PRP as a matrix in which to embed MSCs for cleft palate repair: one group considered PRP a factor contributing to inadequate bone regeneration 94, 95, whereas another group achieved adequate bone regeneration with PRP 96.

It is also known that higher passage number alters MSC potency and efficacy 113, but this consideration must be balanced with the use of cryopreserved versus fresh cells 114. A small study of MSC therapy for acute GvHD found that early passage number was associated with better outcomes, reporting 75% and 86% 1‐year survival and response rate, respectively, in those who received low‐passage MSCs versus 21% and 36%, respectively, in those who received high‐passage MSCs. However only 9 of 31 subjects were children, and they did not report outcomes for children separately; outcomes were similar when they excluded children from the analysis 115. The studies included in this review vary markedly in passage number (from 0 to 9), and, particularly in commercial products (e.g., PNEUMOSTEM and Prochymal), these details are lacking. Use of population doubling time instead of passage number would at least bring some degree of objectivity to this parameter.

Finally, efficacy may be affected by preconditioning, such as hyperoxia exposure 116, highlighting yet another area in need of optimization. Effects of hypoxic preconditioning, inflammatory stimulation, and three‐dimensional culture conditions were recently reviewed 117.

#### MSCs as Delivery Vector

Much of the literature has focused on the MSC's ability to terminally differentiate or secrete immunomodulatory factors, but clinicians in Spain used a highly novel approach taking advantage of the MSC's ability to engraft into tumors. They used BM‐MSCs to deliver the oncolytic adenovirus, ICOVIR‐5, to patients with high‐grade neurologic malignancies 118, 119. At the time of publication, no serious adverse effects were reported, and although only one out of five patients experienced clinical benefit (a 2‐year‐old male with metastatic neuroblastoma), he remained in complete remission 36 months after therapy. The overall clinical benefit was not universal in these studies, but the short‐term clinical safety of MSC therapy was supported.

#### Noncell Therapy

If MSCs can be expanded and differentiated ex vivo, the possibility of using MSC‐conditioned media then exists, which practically eliminates the concern over tumorigenicity. Although MSCs do not appear to be tumorigenic like induced pluripotent stem cells, they may inhibit host antitumor immunity and are implicated in at least one case report of MSC therapy‐related leukemia 120. MSC‐conditioned media and MSC extracellular vesicles have been the subject of several preclinical studies, but these modalities have yet to be trialed in the clinical setting. A complete analysis is beyond the scope of this review, but a brief discussion of several interesting studies follows.

As mentioned above, MSCs secrete numerous growth factors and cytokines into the cell culture media in which they grow; these factors form the “MSC secretome” 121. Use of MSC‐conditioned media in animal models was recently reviewed 122. For example, conditioned media can improve hyperoxia‐induced alveolar and pulmonary vasculature simplification 6, 123, airway hyperreactivity 124, and pulmonary hypertension 123, 124 for up to 6 months in animal models of bronchopulmonary dysplasia.

In addition to soluble factors, MSCs release extracellular vesicles, which are 40‐nm to 1,000‐μm, membrane‐bound bodies containing nucleic acids, proteins, and lipids, and they mediate intercellular communication. Exosomes are a subset of extracellular vesicles ranging from 40 to 150 nm, and microvesicles range from 100 to 1,000 nm 117, 125, 126, 127. A position paper by the International Society for Extracellular Vesicles was recently published 128, including applications of regenerative medicine and regulatory, safety, and manufacturing considerations. Also recently, Akyurekli et al. conducted a systematic review of preclinical studies of microvesicles in animal models of organ injury, tumor growth, or immunomodulation 126. For example, microvesicles ameliorate the inflammation and pulmonary edema induced by intratracheal delivery of lipopolysaccharide in a murine model of acute lung injury 129.

### Important Donor and Recipient Factors

#### Donor

It is known that significant variability between donors exists, but which donor factors affect efficacy is relatively unknown. Particularly in pediatrics, donor age may be an important factor because MSCs from younger donors appear to have greater viability, proliferative potential, and antioxidant capacity 130. As discussed above for BM‐MSCs, adult donors yield “less fit” cells with less proliferative capacity 29, 30, but this must be weighed against the ethics and feasibility of obtaining MSCs in sufficient quantities from younger donors. Also, in many studies, male donors were used with female recipients so as to detect MSC engraftment, but there is some evidence suggesting donor sex may impact MSC phenotype 131. Finally, ABO blood type may impact donor choice, because preliminary studies suggest that UCB‐MSCs from individuals with blood type O have greater proliferative potential and self‐renewal capability than from blood types A or B 132.

#### Recipient/Host

There may be underlying genetic factors dictating response to MSC therapy. Initial investigations toward elucidating these factors were conducted in a trial of UCB‐MSCs for cerebral palsy in twins 83. Significant improvements in gross motor function were observed, and these improvements were highly correlated between individuals of an identical twin pair, but not between twin pairs. In the trial of MSCs as oncolytic adenovirus delivery vectors for neuroblastoma, only one of four children responded 118. Each tumor is unique and consists of a heterogeneous cell population whose members may differentially respond to any therapy; it is possible that only some cells were susceptible to the adenovirus. Clearly, significant research must be conducted to determine which factors or genetic predispositions enhance or diminish MSC efficacy.

#### Allogeneic Versus Autologous/Syngeneic MSCs

It is unknown whether allogeneic or autologous/syngeneic MSC therapy is optimal, and the question of what role the host immune system must play must be answered; these considerations have been previously discussed 133, 134. It is generally believed that MSCs are immune‐evasive, evading lymphocytes by virtue of weak expression of MHC class I and absent MHC class II markers 11, and that one of their main mechanisms of action is immunomodulation. Of course, one benefit of autologous over allogeneic MSC therapy is safety, particularly in the vulnerable pediatric population, but again, quantity and timeliness might make this unrealistic.

The immune‐privileged or immune‐evasive nature of MSCs has been the subject of many preclinical studies. In a murine model of myocardial infarction, Huang et al. found differentiation increased MSC immunogenicity, and specific antidonor antibody developed in recipients: inducing differentiation into myocytes or endothelium resulted in elevated expression of immunogenic markers (MHC‐Ia, MHC‐II, and CD86) and reduced immunomodulatory MHC‐Ib expression 135. However, in a swine model of myocardial infarction, the administration of intracardiac MSCs resulted in minimal development of antidonor antibodies, and there was no detectable antibody‐mediated cytotoxicity 136. Conversely, in healthy rats, prophylactic administration of allogeneic BM‐MSCs induced sufficient immune response to decrease survival of allogeneic MSCs given 2 weeks later 137. A recent review discussed the immunogenic potential of allogeneic MSCs 134, and the data were inconclusive on whether MSCs truly induce T‐cell *allo*‐immunity, as well as their mechanism of immune support. Additionally, MSCs show low engraftment rates and are not retained in vivo; few, if any, minor adverse effects related to treatment have been reported. With the obvious transient nature of the MSCs in vivo, it remains to be determined whether MSC activation and elimination are related to clinical efficacy, which adds to the complexity of MSC therapeutics. Furthermore, as described by Bárcia et al., the complexity extends also in the type of MSC utilized. In the studies of Bárcia et al., compared with BM‐MSCs, MSCs derived from umbilical cord induced less lymphocyte proliferation, while at the same time enhanced the production of regulatory T cells and exhibited a greater anti‐inflammatory effect 138. Although the attention to the details in MSC therapeutics remains in terms of improving optimization of MSCs, these studies suggest that the specific clinical indication and disease phenotype must also be defined to optimize ex vivo manipulations of MSCs to maximize clinical efficacy.

Interestingly, in clinical trials of allogeneic MSC therapy, no significant “rejection” has been reported, although lack of efficacy might be the singular manifestation of “rejected” MSC therapy; better outcomes have been reported for HLA‐matched UCB‐MSC therapy for pediatric cerebral palsy 60. Conversely, in the adult POSEIDON (Percutaneous Stem Cell Injection Delivery Effects on Neomyogenesis) study of allogeneic versus autologous BM‐MSCs for ischemic cardiomyopathy, patients receiving autologous MSCs experienced more frequent treatment‐emergent serious adverse events, although this did not reach statistical significance, but also significant improvement on functional and quality‐of‐life measures 97. Investigators monitored MHC *allo*‐antibody formation up to 6 months after treatment: only one patient developed donor‐specific MHC class I *allo*‐antibodies, but the clinical significance of this is uncertain. Future trials should use similar‐immune monitoring studies to better define the clinical significance of these effects (e.g., correlation with therapeutic efficacy or serious adverse events).

Significantly, the first phase III clinical trials of allogeneic MSC therapy have been completed and are pursuing approval in the U.S. TiGenix completed a European phase III study of Cx601, an allogeneic ADSC product, to treat complex perianal fistulas in adult patients with Crohn's disease, an inflammatory bowel disorder, and a U.S. phase III study is planned for 2017 135. MEDIPOST completed a Korean phase III study of CARTISTEM, an allogeneic UCB‐MSC product, to treat degenerative osteoarthritis in adult patients. CARTISTEM is approved for marketing in Korea by the Korea Ministry of Food and Drug Safety, and a United States phase I/IIa study is being planned 136. Osiris Therapeutics completed phase III studies of Prochymal, an allogeneic BM‐MSC product, for acute GvHD and for severe liver and gastrointestinal GvHD. It is currently approved for treatment of pediatric steroid‐resistant acute GvHD in Canada, and U.S. Food and Drug Administration (FDA) approval is being sought 137. Finally, JCR Pharmaceuticals and MEDIPAL Holdings Corporation announced that TEMCELL HS, an allogeneic BM‐MSC product, has been approved for acute GvHD by the Japanese Ministry, Labor and Welfare 138.

### Practical Considerations

#### Regulations

MSCs and cell‐based therapies are unlike traditional pharmaceuticals by virtue of their living nature, complex physiologic effects, and variability, and therefore require specialized regulation. In the European Union (EU), they are categorized as Advanced Medicinal Therapy Products and are regulated under EU regulation 1394/2007. Exploring cell therapy regulation worldwide is beyond the scope of this review, but we will briefly discuss regulation in the U.S. and refer the reader to several excellent recent articles 139 for international guidelines.

The U.S. FDA Center for Biologics Evaluation and Research (CBER) regulates human cellular and tissue products (HCT/Ps), including stem cell and combination products, under Title 21 of the Code of Federal Regulations Part 1271 (21 CFR 1271), which addresses donor eligibility and testing, current Good Tissue Practices (cGTP), FDA inspections, and requirements for reporting adverse reactions to HCT/Ps. Although current Good Manufacturing Practices (cGMP) are designed to ensure that pharmaceuticals are safe, effective, and pure, cGTP are expressly focused on minimizing transmission of communicable disease. The U.S. FDA/CBER is also a member of several international groups 140, 141 with the goal of standardizing technical guidelines and regulatory requirements to achieve the common public health goal of safe and effective therapies.

The steps required to bring an HCT/P to the bedside differs depending on whether the HCT/P is a “361 product” or “351 product,” based on which section of the Public Health Service Act they fall into. HCT/Ps may be categorized as “361s” only if they meet all of the following criteria (21 CFR 1271.10(a)):

1. The HCT/P is minimally manipulated;
2. The HCT/P is intended for homologous use only, as reflected by the labeling, advertising, or other indications of the manufacturer's objective intent;
3. The manufacture of the HCT/P does not involve the combination of the cells or tissues with another article, except for water, crystalloids, or a sterilizing, preserving, or storage agent, provided that the addition of water, crystalloids, or the sterilizing, preserving, or storage agent does not raise new clinical safety concerns with respect to the HCT/P; and
4. Either:
  - The HCT/P does not have a systemic effect and is not dependent upon the metabolic activity of living cells for its primary function; or
  - The HCT/P has a systemic effect or is dependent upon the metabolic activity of living cells for its primary function, and is for autologous use, is for allogeneic use in a first‐degree or second‐degree blood relative, or is for reproductive use.

It is critically important to determine into which category a cell‐based therapy falls. The 361s are subject to less rigorous regulation and are not required to undergo the extensive premarket approval process, nor are they required to follow cGMP. The 351s are subject to the traditional drug review process, including Investigational New Drug application, preclinical studies, multiphase clinical trials, and Biologics License Application. Unfortunately, many “stem cell clinics” have opened in the U.S. and abroad, liberally interpreting the terminology in 21 CFR 1271.10(a) to avoid this expensive and time‐consuming process 142, 143. They claim to offer autologous, “minimally manipulated,” “homologous” adipose tissue‐derived stem cell therapy meeting FDA standards, and, in response, the FDA has published several “Draft Guidance” documents to begin clarifying this issue 144, 145, 146.

Minimal manipulation is defined as “processing that does not alter the relevant biological characteristics of cells or tissues” (21 CFR 1271.3(f)), such as centrifugation and cryopreservation. However, processing of lipoaspirate to isolate ADSCs is generally considered more than minimally manipulated because it alters the original characteristics of fat tissue. Additionally, homologous is defined as “the repair, reconstruction, replacement, or supplementation of a recipient's cells or tissues with an HCT/P that performs the same basic function or functions in the recipient as in the donor” (21 CFR 1271.3(c)). However, stem cell clinic ADSC treatments are not homologous because they are advertised to treat an enormously wide variety of diseases (e.g., Parkinson's disease, diabetes, lupus, “age management,” etc.) 144.

We are not aware of any clinic in the U.S. that provides these treatments to children, but multiple facilities abroad offer treatment for children as young as 2 years old. Furthermore, we are not aware of any official legal action being taken against these clinics, internationally or domestically, and we support statements opposing these clinics such as the International Society for Cellular Therapy white paper 147 and the International Society for Stem Cell Research Guidelines for Stem Cell Research and Clinical Translation 148.

#### Manufacturing Considerations

Broadly speaking, current GMP strictly regulates all reagents, equipment, and facilities involved in the manufacture of cell‐based therapies 149 and also defines procedures and controls 150. In the U.S., GMP is defined in Title 21 of the Code of Federal Regulations Parts 210 and 211, and in the European Union, by Volume 4 of the EudraLex. The goal is to provide a stable, contaminant‐free, and pure product of known composition and identity 151.

The physical facility setup to minimize risk of contamination involves a combination of clean rooms and cabinets. Use of closed automated devices allows not only for automated inoculation and harvesting, but also bioreactor placement within a less‐stringently aseptic cleanroom 152. Additionally, personnel must have the education, training, and experience required to perform and supervise the manufacturing and processing of the product (21 CFR 211.25).

Contamination by xenobiotic pathogens may be minimized by using only human‐derived supplements (e.g., platelet lysate) and/or by using recombinant growth factor supplements 152, although this is difficult because the optimal cell culture media and/or growth factors have not yet been identified 150. Furthermore, such a change in cell culture conditions may significantly alter MSC qualities 152, 153, requiring further potency testing. All reagents should be screened for infectious agents and communicable diseases through culture for bacteria, fungi, and other agents (e.g., *Mycoplasma*) and be certified free of endotoxin 149, 151, 152. Autologous platelet lysate may be used or be provided as a pooled “off‐the‐shelf” product. In the pediatric setting, one option may be to use autologous MSCs (e.g., UC‐MSCs) expanded ex vivo in allogeneic platelet lysate, limiting the volume of the child's blood needed.

Malignant transformation has not yet been observed in humans who have received MSC therapy, but concerns about genetic stability and tumorigenic potential remain. This is particularly important in pediatrics, where recipients may have many decades of life, if not an entire lifespan, ahead of them. Assays such as karyotype, fluorescent in situ hybridization (FISH), or comparative genomic hybridization (CGH) array should be used, although each has limitations, such as low sensitivity or difficult technique 152. Using only “young” MSCs may reduce the risk of mutations 150, and harmonizing nomenclature by reporting population doubling time rather than passage number may also help identify rapidly expanding transformed clones 153, 154. One approach is suggested by the European Regulatory Authorities: perform conventional karyotype, followed by CGH or FISH analysis if recurrent abnormalities are found 154. Alternatively, polymerase chain reaction assays for genes related to cell cycle control and senescence, as well as oncogenes, can be easily performed 150.

For allogeneic cells, a cell manufacturer must decide on either a “one donor‐one batch” or a master cell stock approach 153. Using a master cell stock may reduce variability between batches and enables quality testing on a proportionately smaller fraction of material. However, developing a master cell stock is expensive, and safety issues (e.g., microbial contamination) found in the master cell stock may result in adverse effects in many recipients. To that end, patient registries ought to be created, allowing monitoring of safety and efficacy, as well as enabling further research.

The product being delivered must be of sufficient purity, and this will likely require specific immunophenotyping 153. Markers such as those established by the International Society for Cellular Therapy 18 are a start, but identifying markers or functional assays to define MSC type (e.g., UC‐MSCs vs. ADSCs) specifically will be needed 153. For example, measuring levels of cytokines and growth factors in the culture media may be appropriate 150, and Nordberg and Loboa discuss such techniques as Raman spectroscopy or electrical impedance spectroscopy to monitor bone or lipid formation or mass spectrometry to assess expansion and differentiation 155. In any case, cryopreservation will likely be required to ensure that the required tests are able to be carried out 150. Finally, any significant changes to the process will require these safety and efficacy tests to be repeated, and may require “upstream” testing, such as pharmacodynamic studies in large animal models of disease or pharmacokinetic dose‐optimization studies and tumor monitoring in immunodeficient mice 156.

#### Facilities and Staff

In addition to cGMP/GTP‐compliant facilities and personnel, a clinical research infrastructure is required. Physicians and staff must be specially trained to screen potential donors and recipients, enroll patients in clinical trials, and provide adequate long‐term follow‐up. Treatment with MSCs will likely require highly specialized providers, so the need for training and experienced medical centers to oversee the procedure will persist for the foreseeable future. For medical trainees, specialization in HCT/P therapy may be added to any program of graduate medical education. Individuals treated as children or infants may require long‐term follow‐up, spanning pediatric and adult medicine, similar to current patients with congenital heart disease or childhood cancers.

Dedicated social workers and Ronald McDonald House‐type facilities to minimize financial costs for families of children receiving HCT/P therapy may be considered to maximize social justice and accessibility, regardless of socioeconomic status. This support should be available during both clinical research trials, as well as during “routine” clinical care that may be provided as standard‐of‐care in the future.

The types of medical centers participating in clinical trials or providing therapeutic HCT/Ps must also be decided on to ensure accessibility. The Management of Myelomeningocele Study (MOMS) provides an enlightening case study 157: MOMS was an 8‐year study “to evaluate the safety and efficacy of prenatal repair of myelomeningocele with that of standard postnatal repair,” during which time all fetal surgery centers in the U.S. except the three study centers agreed not to perform prenatal repair of myelomeningocele. Expectant mothers were randomized to one of three fetal surgery centers, and families were required to cover costs of transport and lodging and be able to take up to 3 months off from work. Therefore, the study population was not representative of the general U.S. population, limiting generalizability. Concentration of expertise and infrastructure within a few major medical centers must be balanced with social justice and accessibility in community centers.

#### Organizational Framework

Developing cell‐based therapies requires a new model to bring these therapies into the clinic because of the complexity of living and heterogeneous cells, the need for GMP facilities and scientific expertise at the point‐of‐care, the need for long‐term follow‐up, and the disease‐specific and patient‐specific modifications of the product 158. Public funding combined with GMP facilities and scientific expertise of academic medical centers, working in collaboration with governmental agencies to navigate regulations and bring awareness to the greater public, will be needed. The business model of in vitro fertilization clinics may be applicable to cell therapy clinics, addressing such topics as insurance reimbursement. By having a limited number of specialized centers, the full breadth and depth of expertise needed will be concentrated and developed, again, balancing these demands with accessibility.

## Conclusion

The central question to MSC therapy is not, “Are MSCs therapeutically effective?” Rather, it is: “How can we optimize MSC therapy for efficacy while avoiding adverse effects?” The breadth of clinical and preclinical investigations must be matched with an equal depth of preclinical studies. It is unlikely that a single type of cell and delivery system will be usable for the wide variety of pathologies amenable to MSC‐based therapy. The purpose of this review is to stimulate future preclinical and clinical studies in all targetable diseases, and to better define the factors that must be optimized to result in maximal therapeutic efficacy.

We must be cautious before broadly adopting the “revolution” that cell‐based therapy represents. Negative results from as well‐designed, definitive clinical trial of MSC therapy for any disease would deal a significant blow to the entire community of stem cell researchers, and preclinical optimization of protocols addressing some of the factors identified in this review will aid in minimizing the chance of such an outcome. Additionally, if introduced too rapidly, revolutionary therapies may result in regret if adverse effects are only appreciated many years later. To this end, stem cell tourism must be discouraged so as to avoid casting the entire field in an unfavorable light.

**Figure 1:**
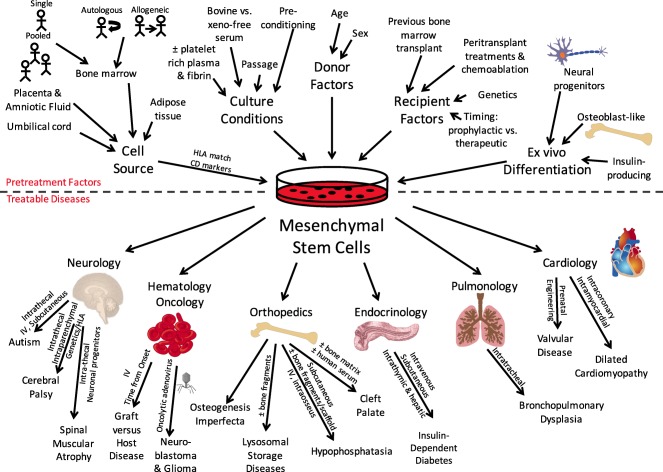
Mesenchymal stem cells have been used in a variety of clinical studies of pediatric diseases, applying to neurology, hematology/oncology, orthopedics, endocrinology, pulmonology, and cardiology. Before large‐scale translation into the clinical arena, however, factors such as cell source, culture conditions, donor factors, recipient factors, and ex vivo differentiation must be addressed. Abbreviations: CD, cluster of differentiation; HLA, human leukocyte antigen.

The wide variety of diseases; sources of MSCs; route, dose, and timing of administration; ex vivo culture conditions and differentiation; and donor and recipient factors (as outlined in Fig. 1) make determining the optimal cell‐based therapy challenging. We need to develop and optimize protocols and markers to evaluate potency and efficacy to better define the phenotype of the MSC for each clinical indication. Patient‐to‐patient variability in the context of a given clinical entity will also likely confound the pursuit of the optimized therapeutic algorithm. Outlining the pretreatment factors of the MSCs and defining the treatable diseases as the outcome is essential to bring this exciting therapy to the bedside.

In the end, it is the investment of time, energy and attention to detail that will optimize the therapeutic application of MSCs in the pediatric population. Pushing this threshold of therapeutic intervention with the potential of medical breakthroughs highlights the multifaceted and innovative potential of cell‐based therapy for pediatric diseases that are currently difficult to treat.

## Author Contributions

C.R.N. and T.L.B.: manuscript writing, final approval of manuscript.

## Disclosure of Potential Conflicts of Interest

The authors indicated no potential conflicts of interest.
